# Development of reference genes for RT-qPCR analysis of gene expression in *Pleurotus pulmonarius* for biotechnological applications

**DOI:** 10.1038/s41598-023-39115-4

**Published:** 2023-07-29

**Authors:** Taísa Godoy Gomes, Fernando Campos de Assis Fonseca, Gabriel Sergio Costa Alves, Félix Gonçalves de Siqueira, Robert Neil Gerard Miller

**Affiliations:** 1 Departamento de Biologia Celular, Instituto de Ciências Biológicas, Universidade de Brasília, Campus Universitário Darcy Ribeiro, Brasília, DF 70910-900 Brazil; 2 Instituto Federal de Goiás (IFG), Águas Lindas, GO 72910-733 Brazil; 3 Embrapa Agroenergia, STN-70297-400, Brasília, DF 70297-400 Brazil

**Keywords:** Reverse transcription polymerase chain reaction, Fungi, Environmental biotechnology

## Abstract

*Jatropha curcas* is an oilseed crop with biorefinery applications. Whilst cake generated following oil extraction offers potential as a protein source for animal feed, inactivation of toxic phorbol esters present in the material is necessary. *Pleurotus pulmonarius* is a detoxifying agent for jatropha cake with additional potential as animal feed, edible mushroom and for enzyme production. For the characterization of fungal genes involved in phorbol ester degradation, together with other industrial applications, reverse transcription-quantitative PCR (RT-qPCR) is a tool that enables accurate quantification of gene expression. For this, reliable analysis requires reference genes for normalization of mRNA levels validated under conditions employed for target genes. The stability of potential reference genes *β-TUB*, *ACTIN*, *GAPDH*, *PHOS*, *EF1α*, *TRPHO*, *LAC*, *MNP3*, *MYP* and *VP* were evaluated following growth of *P. pulmonarius* on toxic, non-toxic jatropha cake and a combined treatment, respectively. NormFinder and geNorm algorithms for expression stability analysis identified *PHOS*, *EF1α* and *MNP3* as appropriate for normalizing gene expression. Reference gene combinations contrasting in ranking were compared following normalization of relative expression of the *CHU_2040* gene, encoding an esterase enzyme potentially involved in phorbol ester degradation. The reference genes for *P. pulmonarius* will facilitate the elucidation of mechanisms involved in detoxification of phorbol esters as well as analysis of target genes for application in biorefinery models.

## Introduction

The current global drive to reduce the negative impacts of industry on the environment has led to the development of the bioeconomy concept, where economic sectors are adapted towards sustainability utilizing renewable biological resources for food, materials and energy production. Agro-industries are undergoing a transformation regarding renewable raw materials, low-carbon production, and land-use optimisation within integrative models such as biorefineries. In this context, *Jatropha curcas* L., an oilseed crop with potential industrial and agricultural applications, is recognized primarily as a potential sustainable biofuel crop^1–3^. Difficulties exist, however, in adding value to the by-product of jatropha cake, which is a lignocellulosic residue rich in proteins, nitrogen, phosphorus, potassium, and carbon. Application of this residue in animal feed is currently limited due to the presence of toxic and thermostable antinutritional factors such as phorbol ester diterpenes^4–6^.

Terpenes are chemical compounds derived from secondary plant metabolism that play a fundamental role in plant development, ecological interactions and defense responses against pests and pathogens^7,8^. These chemical compounds, whilst widely used in industrial processes such as in the production of cosmetics, pharmaceuticals, medicinal products, and insecticides^7,9,10^, are also among the main pollutants accumulating from industrial pulp and paper production^11^. Given this, the bioprospecting of microorganisms capable of degrading such substances and their derivatives is of fundamental importance in bioremediation. The bio-detoxification of diterpene phorbol esters in jatropha cake offers potential for downstream application of the residue as a fertilizer or animal feed supplement. Appropriate micro-organisms may enable not only the degradation of toxic compounds present, but potentially also concomitantly increase nutritional value, or generate other value-added by-products such as enzymes, bioactive compounds and/or edible mushrooms. The genes and metabolic pathways underlying the biodegradation mechanism of phorbol ester in the cake generated after *J. curcas* oil extraction are currently poorly explored. However, it has been demonstrated that specific microbial growth can lead to the secretion of extracellular enzymes such as esterases, lipases, and proteases, which play roles in the degradation of the toxic diterpene^3,12^.

Fungal species of the genus *Pleurotus* are today employed in the bioeconomy, in green chemistry and in the production of edible protein substitutes. These cultivated mushrooms, known as oyster mushrooms, are among the most cultivated globally^13^. Success in production is mainly due to the efficiency of fruiting and cultivation on various substrates^14^. Biotechnological products are numerous, with applications in bioremediation, mycoremediation, biotreatment and enrichment of lignocellulosic waste, biological control, development of catalytic products and in production of polysaccharides such as ergosterol^15–18^. Among the oyster mushrooms, *Pleurotus pulmonarius* is efficient in the degradation of phorbol esters in jatropha cake, with additional potential for application in biorefinery models^3^. The identification of genes in this basidiomycete fungus that are involved in the degradation of phorbol esters has yet to be conducted and represents an important step towards efficient enzymatic degradation and solving bottlenecks related to biodegradation. In this context, high-throughput sequencing approaches for analysis of the fungal transcriptome will be appropriate for the identification of expressed genes and metabolic pathways activated during the degradation of this toxic compound, as well as additional genes encoding and regulating the expression of enzymes with diverse industrial application^3,12^.

For accurate quantification and validation of gene expression data originating from high-throughput transcriptome sequence data, reverse transcription-quantitative PCR (RT-qPCR) is a benchmark analytical tool for application across different experimental conditions^19–24^. Robust analysis, however, requires the employment of a suitable set of reference genes for transcript expression normalization, correcting data for variation across samples. In accordance with the Minimum Information for Publication of Real-Time Quantitative PCR Experiments (MIQE) guidelines^25^, normalization of expression of each target gene in relation to a reference gene is required to correct data for potential variations across sample replicates that often occur during cDNA preparation. For such normalization, specific reference genes are necessary for each organism that consider microbial growth conditions, tissue type and expression time points^25–27^. A suitable reference gene for gene expression normalization should exhibit constant expression, independent of the sample, condition, treatment, cell, and tissue type^28,29^. To determine the most stable normalizing genes, different algorithms with distinct statistical methods are generally applied, which will determine the best sets of stable genes^30–32^.

The genetic stability of reference genes has previously been investigated in different species of the genus *Pleurotus*, including *Pleurotus ostreatus*^19,33–35^; *Pleurotus eryngii*^36^, and *Pleurotus tuber-regium*^37^. Given the potential in *P. pulmonarius* for both the degradation of phorbol esters and concomitant application in biorefinery models using jatropha cake as carbon source, analysis of fungal gene expression is warranted. For accurate future analysis of target gene expression, reference genes for RT-qPCR are therefore required for this species. This study investigates the reliability of potential reference genes for *P. pulmonarius* during growth on jatropha cake, in the presence and absence of toxic phorbol esters.

## Results

### Specificity and efficiency analysis of primers

Fifteen candidate genes were tested for specificity and stability as potential reference genes for *P. pulmonarius* cultivated on jatropha cake in the presence and absence of phorbol esters. Primer information for each evaluated gene is described in Supplementary Table S1. Out of the initial 15 candidate genes, five genes, namely *PEP*, *ACTIN2*, *LAC2*, *CHS*, and *α-TUB* did not display specific amplification via RT-PCR and were therefore not subsequently tested via RT-qPCR. For the remaining 10 genes, the specificity of primer amplification for single gene loci was confirmed by analysing melting curves, as shown in Supplementary Fig. S1, with single peaks obtained for each primer pair. PCR amplification efficiency values for the candidate genes ranged from 1.956 to 1.85, following calculation with the software LinRegPCR (Table 1).

**Table 1:** Primer amplification efficiency in RT-qPCR for the candidate reference genes evaluated for normalization of *Pleurotus pulmonarius* gene expression.

Gene	Description	ID	Primer name	PCR amplification efficiency (%) ± SD
*β-TUB*	β-tubulin	117235	*Pleurotus_pulmonarius*_β-TUB	1.956 ± 0.026
*GAPDH*	Glyceraldehyde 3-phosphate dehydrogenase	1090672	*Pleurotus_pulmonarius*_GAPDH	1.924 ± 0.022
*VP*	Versatile peroxidase	JX021525.1	*Pleurotus_pulmonarius*_VP	1.910 ± 0.024
*LAC*	Laccase	AY836675.1	*Pleurotus_pulmonarius*_LAC	1.893 ± 0.023
*ACTIN*	Actin 1	1087906	*Pleurotus_pulmonarius*_ACTIN	1.904 ± 0.026
*PHOS*	Methioadenosine phospharylase	49987	*Pleurotus_pulmonarius*_PHOS	1.860 ± 0.021
*EF1α*	Elongation Factor 1	OX344738.1	*Pleurotus_pulmonarius*_* EF1α*	1.954 ± 0.023
*MNP3*	Manganese peroxidase	1089546	*Pleurotus_pulmonarius*_MNP3	1.945 ± 0.028
*Trpho*	Trefalose peroxidase	EF645805.1	*Pleurotus_pulmonarius*_Trpho	1.850 ± 0.028
*MYP*	Manganese peroxidase	AB353725	*Pleurotus_pulmonarius*_MYP	1.868 ± 0.024

### Analysis of Cq values of candidate reference genes

**Figure 1:**
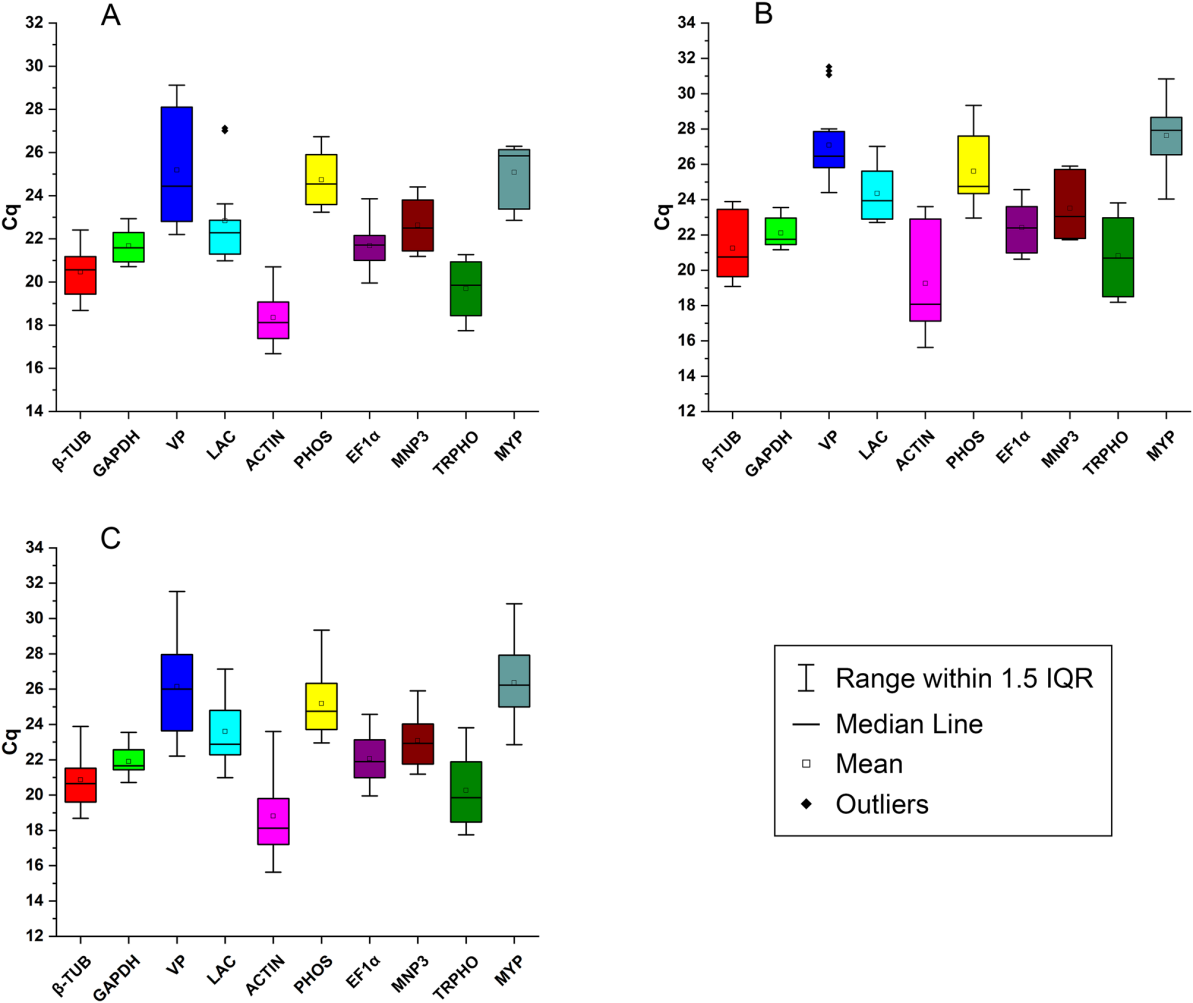
Box plot presentation of the variations in the Cq values for the ten candidate genes tested for stabilizing gene expression of *Pleurotus pulmonarius* cultivated on toxic and non-toxic jatropha cake. (**A**) Toxic, (**B**) non-toxic, (**C**) combined.

Variation in Cq (cycle quantification) values for the 10 candidate genes was analysed and presented using a Box-plot representation, where it is possible to observe expression intervals, average Cq values (mean and median) and discrepant outlier values. Analysis of each gene was carried out under distinct conditions, namely *P. pulmonarius* grown on jatropha cake with the presence of toxic phorbol ester (treatment T) (Fig. 1A), *P. pulmonarius* grown on jatropha cake without toxic phorbol ester (treatment NT) (Fig. 1B), and via consideration of *P. pulmonarius* under both conditions (referred to as a combined treatment (C), representing a dataset of combined T and NT data) (Fig. 1C). In the presence of toxic phorbol esters, Cq values ranged from 16.9 to 29.1 across the candidate reference genes. Under this condition, the *ACTIN* gene presented the lowest number of cycles, indicating greater expression than the other candidate genes, whilst the *peroxidase* (*VP*) gene presented the highest Cq value, indicating lowest gene expression. Greatest variation of Cq was observed for the gene *VP*, with *GAPDH* presenting the lowest variation of Cq, with a mean value and median similar to the *EF1α* gene. The *LAC* gene was the only candidate to present outliers for this condition. For the NT condition of *P. pulmonarius* cultivation on jatropha cake without toxic phorbol esters, Cq values ranged from 15.6 to 30.8. For this condition, the *MYP* gene displayed the highest Cq value, whilst the lowest Cq value was observed for the *ACTIN* gene, where the highest variation of Cq was also observed. The gene *VP* was the only candidate in this condition to present outliers, with the lowest variation of Cq observed for the *GAPH* gene. For the combined condition, *ACTIN* and *VP* genes showed the lowest and highest Cq values, respectively. Both genes, however, showed a large variation in Cq, as also observed for the *MYP* gene. The lowest variation of Cq for this condition was observed for *GAPDH* and *EF1α* genes.

### Expression stability analysis

Stability of candidate reference gene expression stability for *P. pulmonarius* cultivated on jatropha cake representing T, NT and combined data for treatments was assessed using the algorithms BestKeeper, geNorm, and NormFinder. Ranking of expression stability according to each biological treatment is summarized in Table 2.

**Table 2:** Expression stability of the ten candidate reference genes for normalizing gene expression in *Pleurotus pulmonarius* cultivated on jatropha cake.

Condition	Gene	geNorm		NormFinder		BestKeeper	
		Rank	M	Rank	SV	Rank	StdDev [CP ±]
Toxic Jatropha cake	*β-tubulin*	3°	0,139	8°	0,015	_	1.51
	*Glyceraldehyde 3-phosphate dehydrogenase*	7°	0,472	5°	0,014	1°	0.98
	*Versatile peroxidase*	10°	0,642	6°	0,021	_	1.14
	*Laccase*	9°	0,58	4°	0,011	_	1.19
	*Actin 1*	8°	0,522	7°	0,015	_	1.43
	*Methioadenosine phospharylase*	5°	0,317	1°	0,008	_	1.46
	*Elongation Factor*	4°	0,237	3°	0,009	_	1.25
	*Manganese peroxidase*	6°	0,38	2°	0,009	_	1.47
	*Trefalose peroxidase*	2°	0,12	9°	0,015	_	2.11
	*Manganese peroxidase*	1°	0,11	10°	0,016	_	1.46
Non-toxic Jatropha cake	*β-tubulin*	5°	0,335	5°	0,023	_	1.08
	*Glyceraldehyde 3-phosphate dehydrogenase*	8°	0,581	4°	0,020	1°	0.70
	*Versatile peroxidase*	9°	0,789	2°	0,018	_	1.74
	*Laccase*	1°	0,116	9°	0,032	_	1.34
	*Actin 1*	6°	0,394	10°	0,037	_	1.41
	*Methioadenosine phospharylase*	2°	0,118	3°	0,018	_	1.03
	*Elongation Factor*	3°	0,153	1°	0,015	2°	0.94
	*Manganese peroxidase*	4°	0,267	7°	0,029	_	1.23
	*Trefalose peroxidase*	7°	0,494	6°	0,027	_	1.38
	*Manganese peroxidase*	10°	0,988	8°	0,031	_	1.7
Combined	*β-tubulin*	4°	0,446	8°	0,028	_	1.25
	*Glyceraldehyde 3-phosphate dehydrogenase*	7°	0,57	4°	0,020	1°	0.85
	*Versatile peroxidase*	9°	0,731	10°	0,040	_	1.69
	*Laccase*	8°	0,617	7°	0,027	_	1.34
	*Actin 1*	5°	0,499	5°	0,023	_	1.43
	*Methioadenosine phospharylase*	2°	0,323	3°	0,018	_	1.30
	*Elongation Factor*	1°	0,31	2°	0,018	_	1.22
	*Manganese peroxidase*	3°	0,358	1°	0,014	_	1.35
	*Trefalose peroxidase*	6°	0,532	6°	0,026	_	1.84
	*Manganese peroxidase*	10°	0,817	9°	0,032	_	1.49

Results were obtained following analyses with the three distinct algorithms geNorm, NormFinder and BestKeeper. GM, geometric mean; M, average expression stability; SV, stability value; Std Dev, standard deviation; Cp, process capability. When employing NormFinder and geNorm, low stability values indicate greater gene expression stability. When employing BestKeeper, genes with standard deviation values greater than 1 are considered as inconsistent.

**Figure 2:**
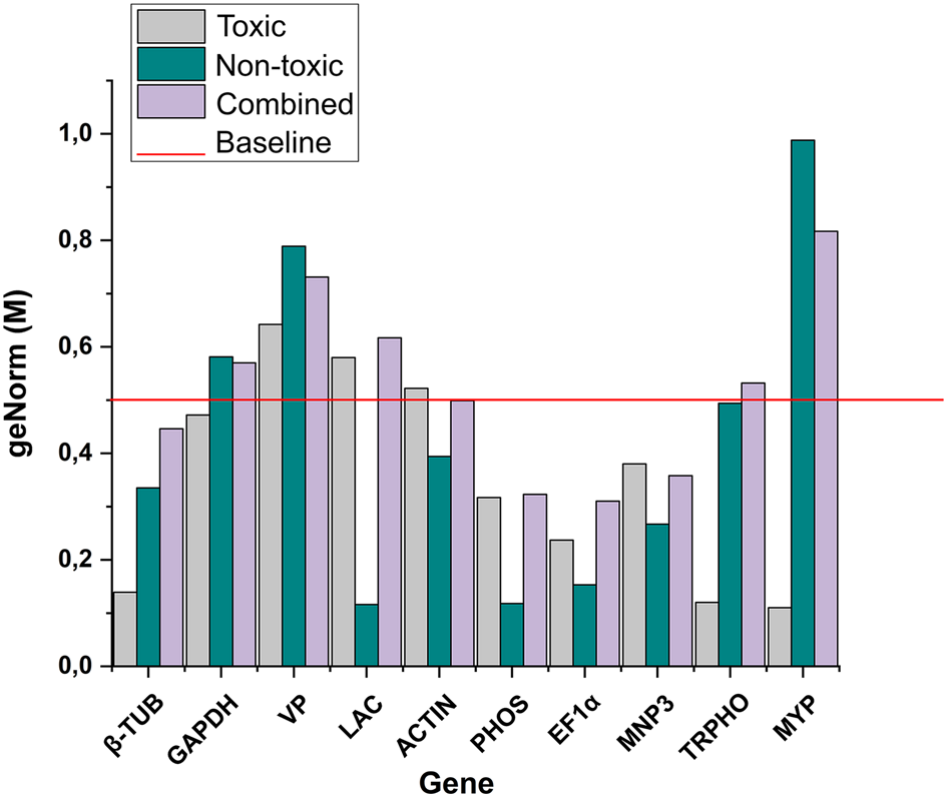
GeNorm algorithm-derived data on the stability of candidate reference genes for normalizing gene expression via qPCR in *Pleurotus pulmonarius* under the three analysed conditions: toxic, non-toxic, and combined. Values of M below a 0.5 cut-off indicate a high stability rate.

**Figure 3:**
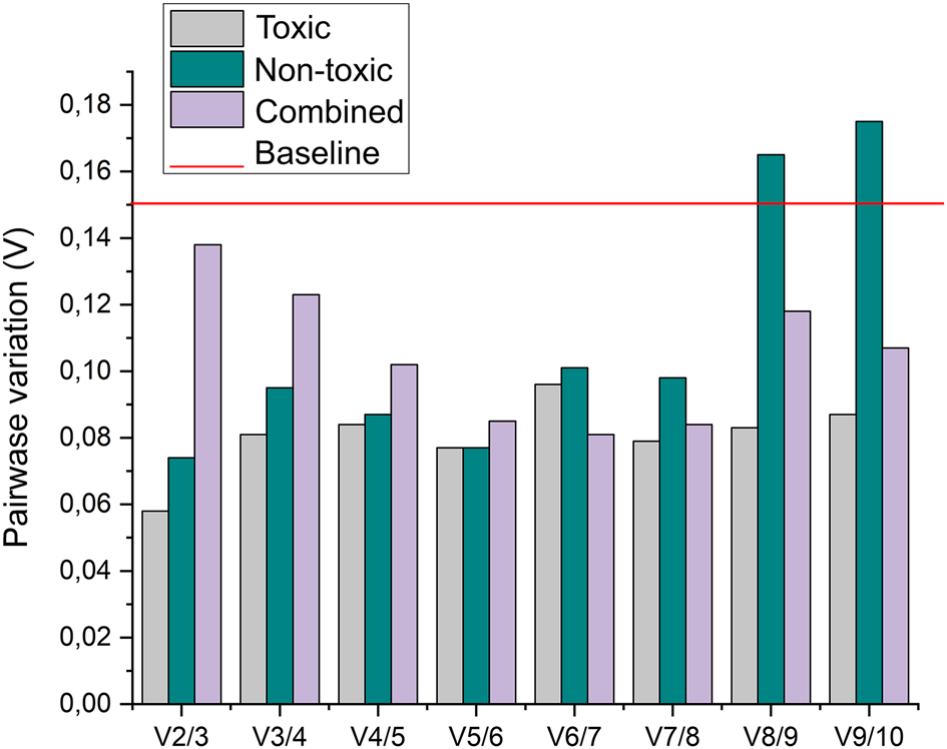
GeNormV-based determination of optimal numbers of reference genes for accurate RT-qPCR normalization of target gene expression. For all data values below a cut-off value of 0.15, additional reference genes do not contribute significantly to gene expression data normalization.

Analysis with the geNorm algorithm revealed stability for the *P. pulmonariu*s candidate genes *β-TUB*, *PHOS*, *EF-1* and *MNP3* across all treatment conditions, with stability values below the default limit of 0.5, representing stability (Fig. 2). In contrast, the *VP* gene showed values above 0.5 across all the treatments. For *MYP*, *TRPHO* and *GAPDH* genes, stability was observed only during fungal growth during treatment T, with values observed of 0.11, 0.12, and 0.472, respectively. The *LAC* and *ACTIN* genes displayed stability only during treatment NT, with values of 0.116 and 0.394, respectively. Pairwise variation analysis (V) was conducted using geNorm to determine the minimum number of genes necessary for normalizing gene expression in *P. pulmonarius* on *J. curcas* cake. Data presented in Fig. 3 shows a pairwise variation value of V2/3 of V < 0.15 for all fungal growth conditions, indicative that two reference genes are sufficient for normalization of gene expression across the treatments.

**Figure 4:**
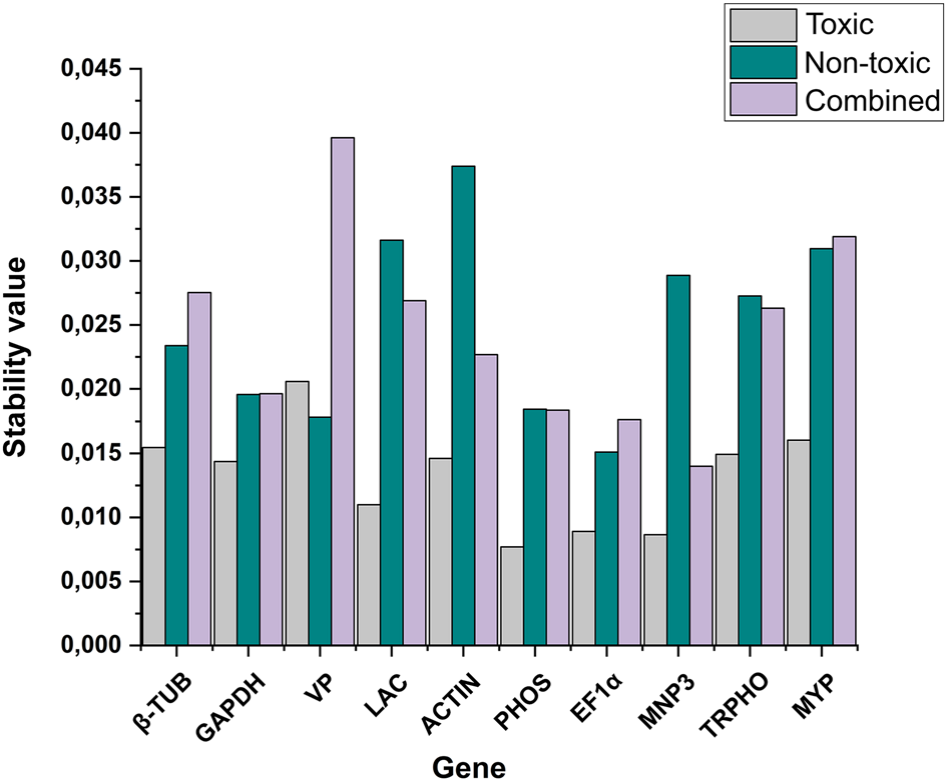
NormFinder algorithm-derived data on the stability of candidate reference genes for normalizing gene expression via qPCR in *Pleurotus pulmonarius* under the three analysed conditions: toxic, non-toxic, and combined. Values of M below a 0.5 cut-off indicate a high stability rate.

Gene stability ranking data following analysis with the algorithm NormFinder is presented in Fig. 4. In agreement with ranking data obtained using geNorm, *PHOS* and *EF1α* genes displayed greatest stability across all treatment conditions. In the case of the *MNP3* gene, analysis with NormFinder indicated high stability in expression in *P. pulmonarius* during cultivation conditions on toxic jatropha cake and when considering data for the combined conditions. Also in agreement with geNorm data, the genes *TRPHO* and *MYP*, displayed greater stability during growth under only the toxic condition, whilst *β-TUB*, *ACTIN* and *LAC* genes, which also displayed stability only during the toxic condition, showed a NormFinder stability profile diverging from geNorm ranking data.

Analysis using the program BestKeeper revealed only the *GAPDH* gene displaying a standard deviation value lower than 1 for *P. pulmonarius* under the three distinct conditions, and the *EF1α* gene displaying a value of 0.94 in *P. pulmonarius* during treatment NT (Table 2). Results were generally discrepant in relation to those obtained with the algorithms geNorm and NormFinder, with the exception of the gene *EF1α*, which showed stability with all three algorithms employed.

### Testing selected reference genes for normalization of a *P. pulmonarius esterase* gene

**Figure 5:**
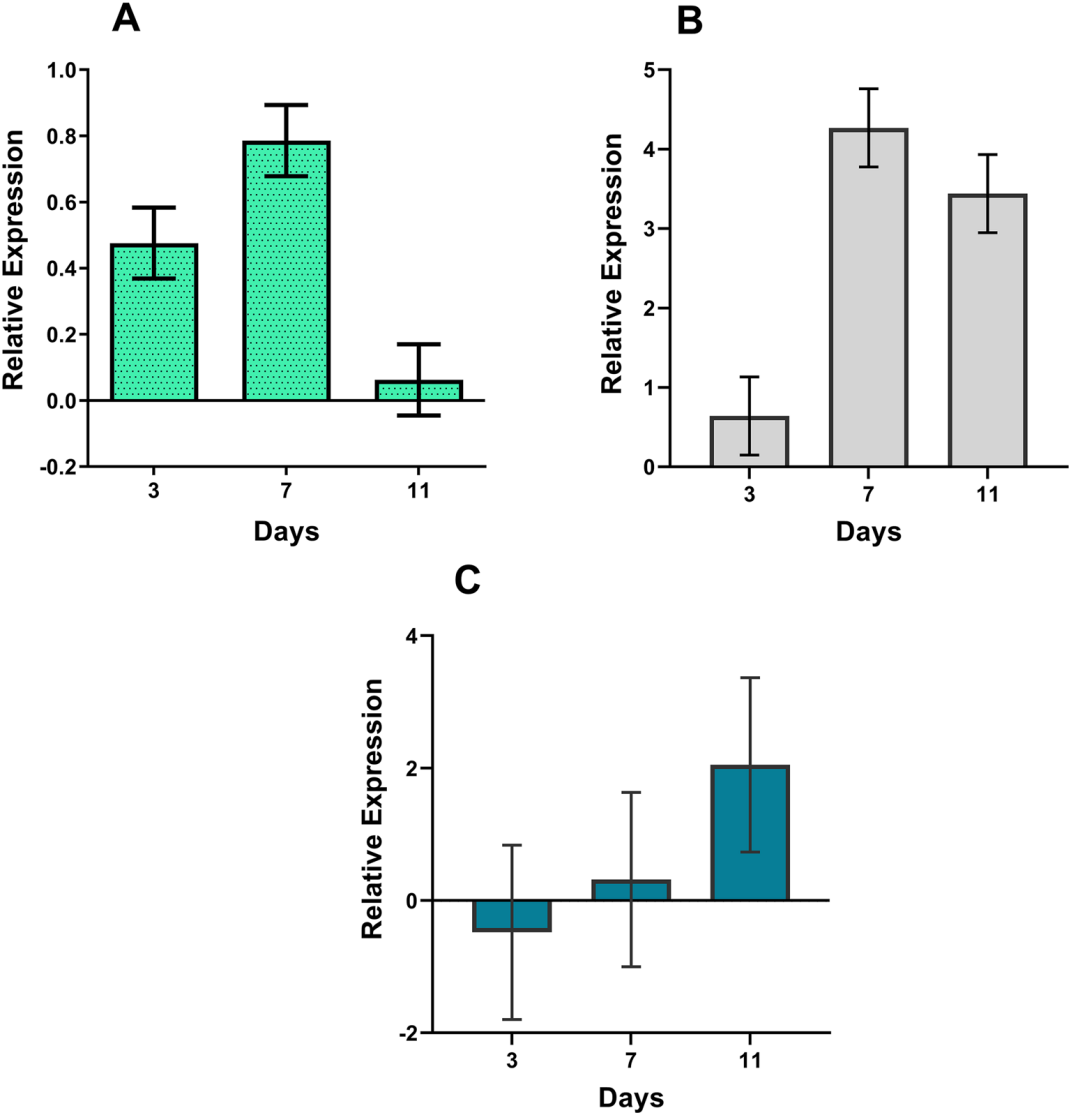
Relative expression levels of the esterase gene *CHU_2040* in *Pleurotus pulmonarius* using the most (*EF1α* and *PHOS*; *EF1α* and *MNP3*) and the least (*LAC* and *VP*) stable pairs of reference genes. RT-qPCR was performed with three biological replicates, considering the combined condition C (datasets for *P. pulmonarius* cultivated on toxic and non-toxic jatropha cake). (**A**) *EF1α* and *PHOS*; (**B**) *EF1α* and *MNP3*; (**C**) *LAC* and *VP*.

To validate reference gene rankings, relative expression of the *CHU_2040* gene was normalized against different reference gene combinations. This gene encodes an esterase enzyme, potentially involved in phorbol ester degradation. Analysis of RNA-seq data from *P. pulmonarius* on *J. curcas* (unpublished), has revealed that the *CHU_2040* gene exhibits differential expression across 3, 7, and 11 days of cultivation on toxic jatropha cake when compared to cultivation on non-toxic cake. For normalization tests using the *CHU_2040* gene, various combinations of genes ranked as most stable (*PHOS* and *EF1α*; *EF1α* and *MNP3*) and as least stable (*LAC* and *VP*) were compared. Figure 5 demonstrates that normalizing the relative expression of the *CHU_2040* gene using the two most stable gene pair combinations revealed a similar positive expression profile of the gene over the time course investigated, albeit with variations in expression levels, and with RT-qPCR data supporting in silico data. Normalization with the two least stable gene candidates, in contrast, resulted in distinct gene expression profiles of the *CHU_2040* gene over the time course, with greater standard deviation.

## Discussion

Given the potential application of *P. pulmonarius* in the degradation of phorbol esters and concomitant enrichment of lignocellulosic residues^3^, the objective of this study was to identify reference genes for normalizing gene expression data for *P. pulmonarius* cultivated on jatropha cake in the presence and absence of the toxic diterpene phorbol ester.

Several potential reference genes were selected for screening in *P. pulmonarius* during growth on toxic and non-toxic jatropha cake. These encoded proteins involved in constitutive fungal activities, such as β-tubulin and Actin 1, which are both involved in structural activity, GAPDH, which is involved in glycolysis, PHOS, that plays an important role in the synthesis of amino acids and nitrogenous bases, EF1α, involved in translation processes, and TRPHO, involved in membrane protection^19,38–40^.

The mathematical algorithms of geNorm^28^, NormFinder^29^ and BestKeeper^41^ have been widely employed for reference gene development across different kingdoms, with combined data generally enabling accurate identification of stable normalizing genes. Here, data obtained using NormFinder and geNorm showed an overlap for the most stable and the least stable genes, in contrast to the results obtained using BestKeeper. Discrepancies in results obtained with the different software are common^19^. BestKeeper is an algorithm that may be inadequate for in vivo samples, since the calculation model applied (geometric mean of reference genes with a standard deviation > 1) does not fit biological system variations and is limited to controlled conditions^23,42^.

Based on the results obtained using geNorm and NormFinder, highest stability among the housekeeping genes evaluated was observed with the genes *PHOS* and *EF1α*. According to Castanera and coworkers^19^, the *PHOS* gene also showed high stability and is suitable for normalizing gene expression in *P. ostreatus* using the same algorithms. The *EF1α* gene was recently introduced as the secondary barcode region for molecular identification of fungi at the species level^43^, including basidiomycetes of the genus *Pleurotus*^44,45^. As such, this gene also adheres to the guidelines for ideal reference genes, with conservation in the target DNA sequence, species specificity, constant copy number, and without allelic variation^36^. Previously, the *GAPDH* gene was reported to be unsuitable for normalizing expression in the basidiomycete *P. tuber-regium*, according to data generated using the geNorm algorithm^37^. Here, the *GAPDH* gene was also shown to be unsuitable for normalizing gene expression in *P. pulmonarius* cultivated on jatropha cake, as was the *ACTIN* gene. Castanera and colleagues^19^ reported that the constitutive genes *ACTIN*, *GAPDH1*, and *β-TUB*, according to results generated using Genorm and NormFinder, were the least stable for normalizing gene expression in *P. ostreatus* cultivated on lignocellulosic residue. Clearly, the use of constitutive genes as reference genes without validation of their stability can lead to errors in gene expression analyses^37,46^. Given the previous reports across the three *Pleurotus* species, the constitutive gene GAPDH does not appear to be adequate for normalizing the expression of genes in basidiomycetes of the genus *Pleurotus*.

*Pleurotus pulmonarius* belongs to the class of saprotrophic basidiomycetes, fungi that have evolved the ability to degrade the complex polymers that make up plant cell walls^47,48^. Within the basidiomycetes, *P. pulmonarius* is classified as a White-rot fungus, capable of completely degrading lignin through the action of oxidative enzymes^48–51^. Laccase (LAC), manganese peroxidase (MnP), lignin peroxidase (LiP), and versatile peroxidase (VP) are important enzymes in lignin degradation and are assumed to be the first in the line of proteins expressed during fungal catabolism of lignin^49^. Members of the genus *Pleurotus* have been reported to possess genes encoding *MNP* and *VP*, but not *LiP*-encoding genes^52^. Given the importance of oxidative enzymes for saprotrophic basidiomycetes, genes encoding LAC, MnP, and VP proteins were also tested for here for gene stability. The literature is scarce regarding the selection of reference genes for *P. pulmonarius*, although the *MYP* gene has been described for QPCR quantification of *P. pulmonarius* mycelia^53^. In our results, the *MYP* gene showed stability only under toxic culture conditions. In addition, this gene showed the highest Cq value in both toxic and non-toxic conditions, suggesting that it may be less expressed in these conditions. The *LAC* and *VP* genes did not show stability during any of the tested conditions, with box-plot data indicating outliers within the analyses. In contrast to the other genes encoding oxidative enzymes, the *MNP3* gene, encoding a distinct manganese peroxidase to that of the *MYP* gene, displayed high stability under the experimental conditions, as did the *PHOS* and *EF1α* genes.

According to Phengnuam and Suntornsuk^54^ and Nakao et al.^55^, esterases are the primary enzymes involved in the degradation of phorbol esters by bacteria of the genus *Bacillus*. Based on in-house RNA-seq data for *P. pulmonarius* on toxic and non-toxic jatropha cake (unpublished), the *CHU_2040* gene encodes an esterase potentially involved in the complete degradation of phorbol esters. Through comparison with RNA-seq data, relative expression of this gene was used to validate the stability of the reference genes. When the most highly ranked stable gene combinations (*PHOS-EF1α* and *EF1α-MNP3*) were applied for normalizing relative expression of the *CHU_2040* gene, differential expression results aligned with the in silico data. In contrast, when the least stable reference genes for normalization were employed, namely *LAC* and *VP*, the expression pattern of *CHU_2040* exhibited a distinct profile that significantly diverged from the in silico expression data, revealing their unsuitability as normalization genes under the specific growth conditions employed for *P. pulmonarius*. These results clearly showed that different reference genes can influence the relative expression of target genes under specific growth conditions.

In summary, this first investigation into the development of reference genes for *P. pulmonarius* identified three stable genes suitable for normalizing gene expression during culture on jatropha cake with or without the toxic compound phorbol ester. In addition, paired variation analyses showed that gene normalization can be performed with two reference genes. Whilst *P. pulmonarius* offers considerable importance in biotechnology, data related to molecular investigation of this fungus is still relatively limited. Together with the recent reference genome for *P. pulmonarius*^16^, the set of reference genes developed here for gene expression normalization is appropriate for future transcriptomic analysis and validation of gene expression of this fungus on this lignocellulosic residue.

## Methods

### Jatropha cake material

Cakes were obtained by mechanically pressing seeds for genetic accessions of non-toxic (accession 169) and toxic *J. curcas* (accession 123), all provided by the germplasm bank at Embrapa Agroenergia. Prior to pressing, seeds were individually heated at 60 °C for two hours using a rotary mixer dryer (SCOLT TECH, ERT 60 II). The pressed seeds were then processed using an oil and grease extractor (SCOLT TECH, SMR 610-6) to obtain both crude oil and the cakes that were employed in this study. Methanolic extraction of phorbol esters and quantification by HPLC was conducted following the method described by Gomes et al.^3^. The concentration of phorbol esters in the toxic cake was measured at 2.17 mg/g, while the non-toxic cake did not contain detectable levels of phorbol esters using the employed technique.

### Fungal material and bioassays

*Pleurotus pulmonarius* strain BRM 055674, originating from the basidiomycete collection at Embrapa Agroenergia, was preserved according to the Castellani method^56^. Subsequently, mycelial disks were transferred to *Jatropha* agar culture medium^3^ and incubated at 28 °C for 7 days. As described above, available naturally non-toxic varieties of *J. curcas*^57^ that do not synthesize or have minimal amounts of phorbol esters were employed in bioassays as controls for analysis of gene expression modulation after fungal growth on toxic jatropha cake. For solid-state cultivation, 100 g of each substrate of toxic and non-toxic jatropha cake were moistened to 70% humidity and transferred to 150 mm × 20 mm Petri dishes. Following sterilization, 7-day old *P. pulmonarius* mycelial plugs were inoculated onto either toxic jatropha cake (treatment T) or non-toxic jatropha cake (treatment NT), then incubated at 28 °C. Fungal mycelia colonizing the substrates were collected at three time points over a 3-, 7- and 11-day incubation period. All bioassays were performed in triplicate. As described above, the third condition analysed corresponded to a combination of T and NT treatment data sets and is referred to as the combined condition throughout (treatment C).

### RNA extraction and cDNA synthesis

Mycelial samples of *P. pulmonarius* were collected from the surface of colonized jatropha cake for both treatments at 3, 7 and 11 days after inoculation (DAI). Mycelia was immediately flash frozen in liquid nitrogen then stored at – 80 °C. Extraction and purification of total RNA for each treatment was performed using an INVITRAP® SPIN PLANT RNA kit (Stratec Molecular GMBH, Berlin, Germany), following the manufacturer’s instructions. Total RNA was treated with DNase I (New England Biolabs, Ipswich, MA, USA) for removal of residual genomic DNA. Total RNA concentration and integrity were analysed via 1% agarose gel electrophoresis and Nanodrop ND-1000 spectrophotometry (Thermo Scientific, Waltham, MA, USA). For synthesis of cDNA, from biological triplicates for each experimental condition (3DAI_NT, 3DAI_T, 7DAI_NT, 7DAI_T, 11DAI_NT, 11DAI_T), a total of 1 μg of total RNA was reverse transcribed to cDNA using Super Script II RT and Oligo(dT) primers (Invitrogen, Carlsbad, CA, USA).

### Primer design and selection

To select potential reference genes with stable expression in *P. pulmonarius* grown on jatropha cake waste, candidate genes were selected for screening that encode proteins likely involved in basal cell activities across diverse species of the genus *Pleurotus* (Supplementary Table S1). The software PrimerQuest Tool (Integrated DNA Technologies—IDT, Iowa, EUA) was employed for the design of specific primers for each candidate reference gene. Expected target amplicons varied from 90 to 120 bp, with each primer pair designed towards predicted exon-exon junctions, in order to avoid amplification from genomic DNA and to ensure amplification from cDNA. The specificity of primer pairs was initially tested by *in-silico* PCR against a local database of RNAseq data for *P. pulmonarius*. Primer specificity and efficiency was then tested against the cDNA originating from the biological triplicates of each experimental condition for *P. pulmonarius* cultivated on toxic and non-toxic jatropha cake.

### Reverse transcription-quantitative PCR

A Platinum SYBR Green qPCR Super Mix-UDG w/ROX kit (Invitrogen, Carlsbad, CA, USA) was employed for RT-qPCR analysis of expression of potential reference genes. PCR amplifications were performed using an ABI StepOne® Real-Time PCR thermocycler (Applied Biosystems, Foster City, USA). PCR amplification was conducted on three experimental replicates and three technical replicates per treatment. Each PCR reaction comprised 2 μL of a 1:20 dilution of template cDNA, 0.2 μM of each primer and 5 μL Platinum® SYBR® Green qPCR Super Mix-UDG w/ROX kit (Invitrogen, Carlsbad, CA, USA), to a final volume of 10 μL. PCR thermocycling comprised 52 °C for 2 min, 95 °C for 10 min, followed by 40 cycles of denaturation at 95 °C for 15 s, followed by primer annealing and extension at 62 °C for 60 s. Analysis of the Tm (dissociation) of amplicons was performed using the software SDS 2.2.2 (Applied Biosystems, Foster City, USA) to verify specificity of primers. Raw ΔRn data was applied to determine RT-qPCR efficiency for each gene by using the program LinRegPCR, version 2017.1 (https://www.gear-genomics.com/rdml-tools/).

### Analysis of expression stability

Stability analysis and validation of expression for each candidate reference gene were based on quantification cycle (Cq) values for each cDNA sample. This value indicates the total number of amplification cycles during the PCR exponential amplification phase that are needed to reach a default threshold value for amplification detection. The expression stability of each gene was determined using the analytical algorithms geNorm^28^, NormFinder^29^ and BestKeeper^41^. GeNorm was also employed to calculate expression stability based on an average M-value, representing the pairwise variation of a particular gene against all other genes. Greatest stability in expression is represented by the lowest M-values, with the most stable genes displaying M-values below a threshold of 0.5. GeNorm also calculates the pairwise variation V-value (Vn/n + 1) between each potential reference gene, for determination of the optimal number of reference genes for employment in gene expression normalization. Subsequently, the software qbase + (CellCarta, Montreal, Quebec) was used to calculate the average quantification cycles (Cqs) per gene and the software GraphPad Prism v7 (Dotmatics, Boston, EUA) for statistical analysis.

### Expression analysis of *CHU_2040*

Combinations of the most stable genes (*PHOS* and *EF1α*; *MNP3* and *EF1α*) and the least stable reference genes (*LAC* and *VP*) were compared for normalization of the relative expression of the *CHU_2040* gene, which encodes an esterase enzyme, potentially involved in phorbol ester degradation. RNA extraction, cDNA synthesis and qPCR were all conducted as previously described. The software qbase + (Biogazelle) was employed to calculate the average quantification cycles (Cqs) per gene, with the software GraphPad Prism v7 employed for statistical analysis of the combined condition.

## Supplementary Information

Supplementary Figure S1.

Supplementary Table S1.

## Data Availability

All data generated and analysed during the study is included in the published article and its Supplementary Information files.
