# Supplementary material for: Development of reference genes for RT-qPCR analysis of gene expression in Pleurotus pulmonarius for biotechnological applications
Source: Sci Rep. 2023 Jul 29;13:12296. doi: 10.1038/s41598-023-39115-4 (PMC10387064; doi:10.1038/s41598-023-39115-4)
Supplement: Supplementary file 1 — Supplementary Figure S1. [file 41598_2023_39115_MOESM1_ESM.docx]

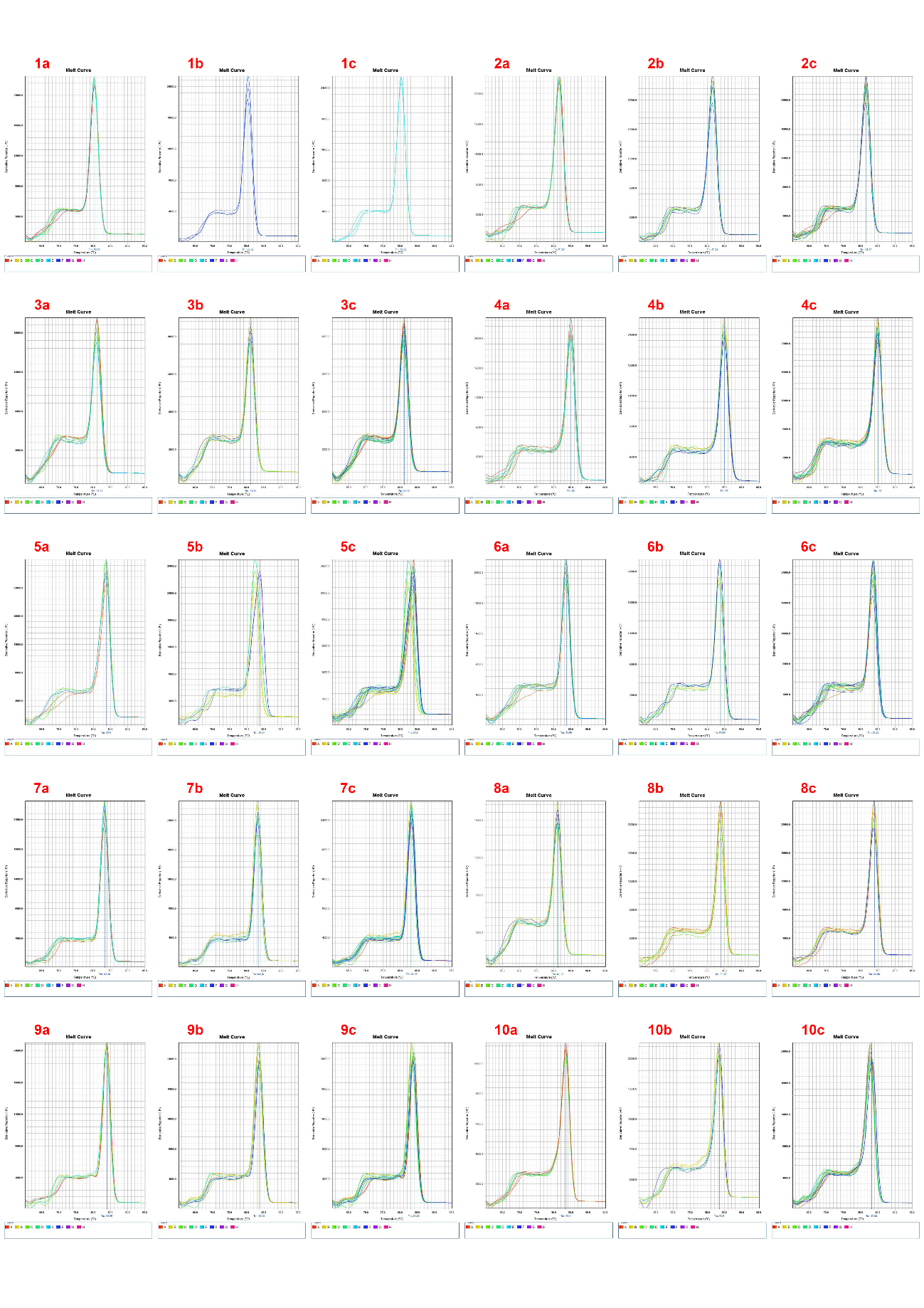


**Figure S1.** Dissociation curves of the candidate reference genes. 1- *β-TUB,* A-Toxic, B- Non-Toxic, C- Combined; 2-*GAPDH,* A-Toxic, B-Non-Toxic, C-Combined; 3-*VP,* A-Toxic, B-Non-Toxic, C- Combined 4-*LAC,* A*-*Toxic, B-Non-Toxic, C- Combined; 5-*ACTIN,* A*-*Toxic, B-Non-Toxic, C- Combined; 6-*PHOS,* A*-*Toxic, B-Non-Toxic, C- Combined; 7-*EF1α,* A*-*Toxic, B-Non-Toxic, C- Combined; 8-*MNP3,* A*-*Toxic, B-Non-Toxic, C- Combined; 9-*TRPHO,* A*-*Toxic, B-Non-Toxic, C- Combined; 10-*MYP,* A*-*Toxic, B-Non-Toxic, C- Combined.
