# Supplementary material for: Development of reference genes for RT-qPCR analysis of gene expression in Pleurotus pulmonarius for biotechnological applications
Source: Sci Rep. 2023 Jul 29;13:12296. doi: 10.1038/s41598-023-39115-4 (PMC10387064; doi:10.1038/s41598-023-39115-4)
Supplement: Supplementary file 2 — Supplementary Table S1. [file 41598_2023_39115_MOESM2_ESM.docx]

**Supplementary Table S1.** Supporting information on candidate reference gene primers for normalizing gene expression in *Pleurotus pulmonarius* in growth treatments comprising cultivation on toxic and non-toxic jatropha cake and combined treatment datasets.

| **Gene** | **Primer sequence (5′-3′)** | **Length (bp)** | **Amplicon size (bp)** | **Tm (°C)** |
| --- | --- | --- | --- | --- |
| *β-TUB* | **F**-GCGATCTCAACCATCTCATCT | 22 | 102 | 62 |
|  | **R**-TGTTGACGGCCAACTTTCT | 19 |  |  |
| *GAPDH* | **F**-CTCCGAGACGACACACAAAG | 20 | 102 | 62 |
|  | **R**-GCGGTAGAGTCAAAGATGGAAG | 22 |  |  |
| *VP* | **F**-CCCATCCTCGATGACATTCAA | 21 | 119 | 62 |
|  | **R**-CCGCCTAAAGTAGGAGAGAAAC | 22 |  |  |
| *MYP* | **F**-CTCTTTGACTCGCAAATCTTCATC | 24 | 121 | 62 |
|  | **R**-GATCGGACTGGAGTCTCATTTC | 22 |  |  |
| *LAC* | **F**-CTCTATCGACGGTCACTCTTTG | 22 | 118 | 62 |
|  | **R**-GGCATTCAACACGAAGGAATAG | 22 |  |  |
| *ACTIN* | **F**-CGTGACCTCACCGATTTCTT | 20 | 109 | 62 |
|  | **R**-CGTAGCACAACTTCTCCTTGA | 21 |  |  |
| *ACTIN2* | **F**-GTTGCTGGTCGTGATCTTACT | 21 | 104 | 62 |
|  | **R**-TCTTTGATGTCCCTCACGATTT | 22 |  |  |
| *PEP* | **F**-GTACCGTTGCATTCCTTCAAA | 22 | 93 | 62 |
|  | **R**-GGCCTTCTTGTCGATCCTAATC | 22 |  |  |
| *PHOS* | **F**-GTGGCTTGTATCACCTCGATAA | 22 | 105 | 62 |
|  | **R**-CAGAAGGGAGAGCACAGATG | 20 |  |  |
| α-TUB | **F**-GAGAGGATACCCGTTCACCA | 20 | 96 | 60 |
|  | **R**-AGCTCCTGCTCAAAGTCCAA | 20 |  |  |
| *LAC2* | **F**-GCCTGGGTTATCTGTCGTAAA | 21 | 108 | 62 |
|  | **R**-GGCTCCACTACGTACAACTTT | 21 |  |  |
| *CHS* | **F**-ATGTACCTTGCCGAAGATCG | 20 | 80 | 60 |
|  | **R**-ATTGCTCCCTGGACATATCG | 20 |  |  |
| *EF1α* | **F**-CGTATCCCTATCGCGAATGT | 20 | 90 | 60 |
|  | **R-**TTTTCCCCGACTTGTACGAC | 20 |  |  |
| *MNP3* | **F-**CTTCGTTAACAACCAAGCCAAG | 22 | 107 | 62 |
|  | **R-**TGACATCGGAGCAGTCAATAAG | 22 |  |  |
| *TRPHO* | **F-**ATGCCTTGATCCGATTCTTG | 20 | 113 | 60 |
|  | **R-**GCCTTGGAGGATGTTGTGAT | 20 |  |  |
